# Supplementary material for: The Cryptococcus neoformans STRIPAK complex controls genome stability, sexual development, and virulence
Source: PLoS Pathog. 2024 Nov 19;20(11):e1012735. doi: 10.1371/journal.ppat.1012735 (PMC11614259; doi:10.1371/journal.ppat.1012735)
Supplement: S8 Fig — The indicated strains were grown overnight in YPD media to saturation. Cells were spread onto YPD plates and allowed to dry before adding fluconazole Etest strips. Plates were incubated at 30°C and images were taken after 48 hours. (DOCX) [file ppat.1012735.s008.docx]

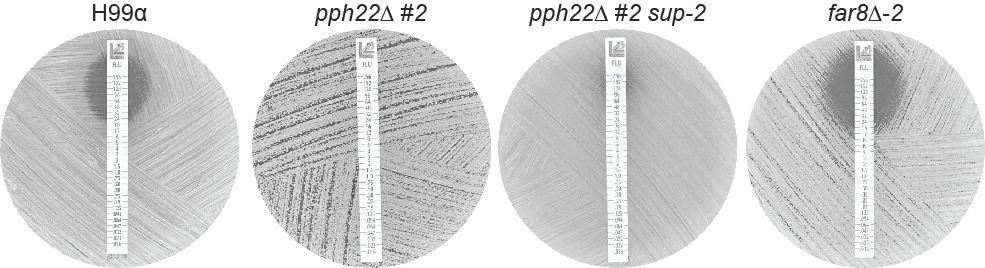


**S8 Fig. Fluconazole Etest of *pph22*Δ, *pph22*Δ *sup*, and *far8*Δ mutants**

The indicated strains were grown overnight in YPD media to saturation. Cells were spread onto YPD plates and allowed to dry before adding fluconazole Etest strips. Plates were incubated at 30°C and images were taken after 48 hours.
