# Supplementary material for: The Cryptococcus neoformans STRIPAK complex controls genome stability, sexual development, and virulence
Source: PLoS Pathog. 2024 Nov 19;20(11):e1012735. doi: 10.1371/journal.ppat.1012735 (PMC11614259; doi:10.1371/journal.ppat.1012735)
Supplement: Supplementary file 2 [file ppat.1012735.s001.docx]

**A.**

**B.** 

**S1 Fig. Protein-protein interactions among STRIPAK components detected by yeast two-hybrid assay.**

(A) Yeast two-hybrid analyses of a 6x6 crossing of strains co-expressing plasmids encoding Gal4 DNA-binding and Gal4 transcription activation domains, fused to STRIPAK complex subunits. Expression of reporter genes *ADE2* and *HIS3*, indicating a positive interaction between GBD and GAD fusion proteins, makes cells appear less red on medium with histidine (Ade+) and able to grow on medium deficient in both histidine and adenine. (B) Confirmation of positive protein-protein interactions on media deficient in histidine and adenine, and on media with 3 mM 3-amino-1,2,4-triazole (3-AT). Autoactivation of the reporter genes in GBD-Pph22 allows for growth on media without histidine.
