# Supplementary material for: The Cryptococcus neoformans STRIPAK complex controls genome stability, sexual development, and virulence
Source: PLoS Pathog. 2024 Nov 19;20(11):e1012735. doi: 10.1371/journal.ppat.1012735 (PMC11614259; doi:10.1371/journal.ppat.1012735)
Supplement: Supplementary file 4 [file ppat.1012735.s003.docx]

**A.** 

**B.**

**S3 Fig. Confirmation of** ***far8*****Δ** **deletion mutants.**

(A) PCR genotyping of the 5’ and 3’ junctions for representative *far8*Δ mutants in the H99α background. WT (H99α) served as a negative control. (B) Snapshot of DNA read coverage at the *FAR8* locus from Illumina whole genome sequencing in a *far8*Δ mutant strain. The coding sequence is depicted in yellow and individual DNA reads are shown below in black. The *FAR8* gene was deleted from the middle of exon 1 to the middle of the last exon (exon 7), as verified by the absence of reads. The read coverage for the indicated chromosomal coordinates is shown in blue.
