# Supplementary material for: The Cryptococcus neoformans STRIPAK complex controls genome stability, sexual development, and virulence
Source: PLoS Pathog. 2024 Nov 19;20(11):e1012735. doi: 10.1371/journal.ppat.1012735 (PMC11614259; doi:10.1371/journal.ppat.1012735)
Supplement: Supplementary file 6 [file ppat.1012735.s005.docx]

**S5 Fig. Serial dilution assay of** ***PPH22/pph22*****Δ** **heterozygous mutant and wild-type diploid strains.**

Wild-type (CnLC6683) and *PPH22/pph22*Δ diploid strains were pre-grown in YPD liquid cultures overnight at 30°C. Cells were then serially diluted and spotted onto YPD medium and incubated at 30°C. The *PPH22/pph22*Δ strains did not exhibit any observable growth defects compared to the wild-type control strain.
