# Supplementary material for: The Cryptococcus neoformans STRIPAK complex controls genome stability, sexual development, and virulence
Source: PLoS Pathog. 2024 Nov 19;20(11):e1012735. doi: 10.1371/journal.ppat.1012735 (PMC11614259; doi:10.1371/journal.ppat.1012735)
Supplement: Supplementary file 7 [file ppat.1012735.s006.docx]

**S6 Fig. AlphaFold structure prediction of the PP2A heterotrimeric enzyme.** Shown with the scaffold subunit Tpd3, regulatory B’’’ subunit Far8, and either Pph22 (left) or Ppg1 (right) as the catalytic subunit. The WD40 and coiled-coil (CC) domains of Far8 are labeled.
