# Supplementary material for: The Cryptococcus neoformans STRIPAK complex controls genome stability, sexual development, and virulence
Source: PLoS Pathog. 2024 Nov 19;20(11):e1012735. doi: 10.1371/journal.ppat.1012735 (PMC11614259; doi:10.1371/journal.ppat.1012735)
Supplement: Supplementary file 8 [file ppat.1012735.s007.docx]

**S7 Fig. Bilateral crosses of** ***pph22*****Δ*****, far8*****Δ*****,*** **and** ***mob3*****Δ** **mutants.**

Strains were grown in YPD overnight cultures before mixing cells in a 1:1 ratio for spotting onto MS medium. Each cross was spotted 10 times onto an MS plate, along with one H99α x KN99**a** wild-type control. Bilateral (mutant x mutant) crosses were incubated at room temperature (24°C) in the dark for 8 weeks. No signs of mating between *pph22*Δ*, far8*Δ*,* and *mob3*Δ strains, in both **a** and α mating types, were observed.
