# Supplementary material for: The Cryptococcus neoformans STRIPAK complex controls genome stability, sexual development, and virulence
Source: PLoS Pathog. 2024 Nov 19;20(11):e1012735. doi: 10.1371/journal.ppat.1012735 (PMC11614259; doi:10.1371/journal.ppat.1012735)
Supplement: Supplementary file 9 [file ppat.1012735.s009.docx]

**S9 Fig. FACS analysis of** ***far8*****Δ mutant strains on RPMI medium.**

*far8*Δ mutants and wild-type 1N (H99) and 2N (CnLC6683) control cells were grown on RPMI medium and stained with propidium iodide to quantify DNA amount. FACS profiles of *far8*Δ cells from RPMI are similar to those from YPD with two major peaks occurring at 2C and 4C, suggesting they are diploid.
