# Supplementary material for: The Cryptococcus neoformans STRIPAK complex controls genome stability, sexual development, and virulence
Source: PLoS Pathog. 2024 Nov 19;20(11):e1012735. doi: 10.1371/journal.ppat.1012735 (PMC11614259; doi:10.1371/journal.ppat.1012735)
Supplement: Supplementary file 10 [file ppat.1012735.s010.docx]

**S10 Fig. Phenotypic analyses of** ***mob3*****Δ mutant strains.**

Serially diluted wild-type KN99**a** and isogenic *mob3*Δ mutants were spotted onto YNB, as well as YPD supplemented with rapamycin, FK506, cyclosporine A (CsA), caffeine, or calcofluor white (CFW). Plates were incubated at 30°C and images were taken after 3 days.
