# Supplementary material for: The Cryptococcus neoformans STRIPAK complex controls genome stability, sexual development, and virulence
Source: PLoS Pathog. 2024 Nov 19;20(11):e1012735. doi: 10.1371/journal.ppat.1012735 (PMC11614259; doi:10.1371/journal.ppat.1012735)
Supplement: Supplementary file 11 [file ppat.1012735.s011.docx]

**S11 Fig. Volcano plot of DEGs in** ***pph22*****Δ** ***suppressor*** **mutants**

Log_2 _fold change is plotted against the -log_10 _*P* value for all DEGs showing significantly upregulated (red) and downregulated (blue) genes. The top 10 most significant genes are labeled.
