# Supplementary material for: The Cryptococcus neoformans STRIPAK complex controls genome stability, sexual development, and virulence
Source: PLoS Pathog. 2024 Nov 19;20(11):e1012735. doi: 10.1371/journal.ppat.1012735 (PMC11614259; doi:10.1371/journal.ppat.1012735)
Supplement: Supplementary file 12 [file ppat.1012735.s012.docx]

**S12 Fig. Heatmaps depicting hierarchical clustering of differential gene expression in** ***pph22*****Δ (*****p*****Δ),** ***pph22*****Δ** ***suppressor*** **(*****p*****Δs),** ***far8*****Δ (*****f*****Δ), and** ***mob3*****Δ (*****m*****Δ) strains compared to wild type (WT).**
