# Supplementary material for: The Cryptococcus neoformans STRIPAK complex controls genome stability, sexual development, and virulence
Source: PLoS Pathog. 2024 Nov 19;20(11):e1012735. doi: 10.1371/journal.ppat.1012735 (PMC11614259; doi:10.1371/journal.ppat.1012735)
Supplement: Supplementary file 13 [file ppat.1012735.s013.docx]

**S13 Fig. Potential direct or indirect targets of the STRIPAK complex.** (A) Expression (log_2 _fold change) of nine genes that are oppositely regulated in *mob3*Δ mutants compared to *pph22*Δ and *far8*Δ*.* (B) Table listing gene ID numbers of the identified genes and their annotations from FungiDB.
