# Supplementary material for: The Cryptococcus neoformans STRIPAK complex controls genome stability, sexual development, and virulence
Source: PLoS Pathog. 2024 Nov 19;20(11):e1012735. doi: 10.1371/journal.ppat.1012735 (PMC11614259; doi:10.1371/journal.ppat.1012735)
Supplement: Supplementary file 16 [file ppat.1012735.s016.docx]

**S3 Table. Differentially expressed genes in** ***pph22*****Δ,** ***pph22*****Δ** ***sup, far8*****Δ, and** ***mob3*****Δ** 

**mutants from RNA sequencing analysis.**

| Comparison | Total DEGs | Up* | Down* |
| --- | --- | --- | --- |
| *pph22*Δ vs. WT | 6944 | 648 (9%) | 230 (3%) |
| *pph22*Δ *sup.* vs. WT | 6964 | 569 (8%) | 299 (4%) |
| *far8*Δ vs. WT | 8280 | 543 (7%) | 359 (4%) |
| *mob3*Δ vs. WT | 7984 | 38 (0.5%) | 44 (0.6%) |

* log_2 _fold change > 1.5 or < -1.5; *p* adjusted value < 0.05
